# Perspectives on voluntary assisted partner notification among providers, people with HIV and the general population in Indonesia: a formative qualitative study

**DOI:** 10.1186/s12889-021-10332-8

**Published:** 2021-01-30

**Authors:** Gede Benny Setia Wirawan, Pande Putu Januraga, I. Gusti Agung Agus Mahendra, Ngakan Putu Anom Harjana, Tiara Mahatmi, Lanny Luhukay, Bharat Bhushan Rewari, Cheryl Johnson, David A. Katz

**Affiliations:** 1 Center for Public Health Innovation, Faculty of Medicine, Udayana University, Bali, Denpasar, Indonesia; 2 World Health Organization Indonesia, Jakarta, Indonesia; 3 Indonesian Ministry of Health, Jakarta, Indonesia; 4 South-east Asia Regional Office, World Health Organization, New Delhi, India; 5 Global HIV, Hepatitis and STI Programme, World Health Organization, Geneva, Switzerland; 6 Department of Global Health, University of Washington, Seattle, USA

**Keywords:** HIV/AIDS, Assisted partner notification, Provider-assisted referral, Index testing, HIV partner services, Indonesia

## Abstract

**Background:**

Voluntary assisted partner notification (aPN) services are effective in increasing access to and uptake of HIV testing among partners of people with HIV. Following recommendations by the World Health Organization in 2016, Indonesia evaluated various approaches to aPN. We present the lessons learned from formative operational research undertaken to understand provider and patient perspectives on aPN from three demonstration sites in cities with a high HIV burden.

**Methods:**

We conducted a formative qualitative study in three cities: Jakarta, Semarang, and Denpasar between September and October 2019. We conducted six focus group discussions (FGDs) (*n* = 44 participants) among health-care providers, people living with HIV and the general population. We explored participant preferences and concerns about how aPN should be delivered, including the methods of and messaging for contacting partners. All FGDs were conducted in the Indonesian language. Qualitative data were analysed using thematic analysis.

**Results:**

aPN was acceptable across different participant populations, although with caveats. Some differences were observed between the general population, providers and people living with HIV. People living with HIV were mainly concerned with confidentiality of the procedure and preferred the use of telecommunication and messages that avoid explicit mention of HIV exposure. Providers preferred similar approaches but for different reasons, being concerned mainly with self-efficacy and security. There was consensus regarding dual referral models. The use of phone calls and short messages were preferred as these are perceived to minimize negative reactions and stigma, protect client confidentiality and are suitable in the current legal situation. The general population was mainly concerned with effectiveness and prefer direct provider-led approaches, such as preferring in-person meeting with explicit notification of potential HIV exposure.

**Conclusions:**

We found consensus among stakeholders on acceptance of aPN, especially dual referral methods. Development and implementation of aPN protocols should also consider clients’ individual situations and concerns regarding safeguarding of confidentiality, and offer a range of options to accommodate all stakeholders involved.

**Supplementary Information:**

The online version contains supplementary material available at 10.1186/s12889-021-10332-8.

## Background

HIV/AIDS remains one of the most fundamental health issues faced by the world. More than 38 million people are living with HIV (PLHIV) and more than 1.7 new HIV infections occur every year. Over half of the new infections are among key populations and their partners [1]. Partners of PLHIV are a priority group in need of HIV testing so that they can know their status, and access HIV treatment and prevention services accordingly [2]. In many settings, however, access to and uptake of testing among partners of PLHIV is poor [3]; in some contexts, as few as one third of PLHIV report that their partners have tested for HIV [4].

Indonesia, with approximately 640,443 PLHIV in 2018, and only 50% of PLHIV aware of their status and 16.9% on antiretroviral therapy (ART), is one of the countries with a fast-growing HIV epidemic [1]. Efforts have been made to increase access to HIV testing, prevention and treatment through integration of HIV care from prevention, testing and treatment through the “Layanan Komprehensif Berkesinambungan” (i.e. continuum of care). However, barriers such as lack of knowledge of HIV, poor risk perception, stigma and suboptimal access to care continue to hinder efforts to increase coverage, particularly for those most affected by HIV [5–9]. To address this gap, in 2019, the Indonesian government prioritized innovations for HIV testing services by initiating the development of a voluntary assisted partner notification (aPN) programme.

Voluntary aPN is a standard practice for many infectious diseases, including sexually transmitted infections, and has been proven to be safe and effective for increasing HIV testing and diagnoses among partners of PLHIV [10]. More recently, two large scale aPN projects conducted in sub-Saharan Africa found aPN is feasible and effective in improving HIV case-finding at scale [11, 12]. Based on available evidence of the acceptability and effectiveness of aPN, WHO recommended aPN in 2016 for all people with HIV as a strategy for reaching PLHIV who do not know their status [13].

Following release of these recommendations, an increasing number of countries have adopted and started to implement aPN approaches. In 2019, over 70% of 136 reporting low- and middle-income countries indicated that they were using this approach and/or had a policy in place to do so [14]. While this marks substantial progress, efforts are still needed to overcome provider- and patient-level barriers and concerns, and optimize implementation to achieve public health impact [15–17].

While the situation is not unique, stigma against HIV is common in Indonesia, including among healthcare workers [18, 19]. Several organizational and cultural factors have contributed to HIV stigma locally, including religious values [19]. Indeed, mistrust of healthcare workers and perceived stigma have been cited as reasons for delaying HIV tests in key populations [5], which has significant implications for the effectiveness of aPN, which relies on acceptability and trust from all stakeholders.

Understanding provider and client preferences in different settings within Indonesia was thought to be critical to developing and implementing a successful aPN programme locally. We aimed to explore the perceptions of aPN among the general population, PLHIV and health-care providers, and identify preferences for and barriers to effective services. In doing so, this study will help inform the development and implementation of a safe, effective and feasible approach to and implementation of aPN across Indonesia and similar settings.

## Methods

### Research settings

Demonstration sites were selected in three high HIV burden cities (Jakarta, Denpasar and Semarang) by the Indonesian government in November 2018, following a brief orientation and training of health-care providers, including two meetings. Health-care providers who underwent training were health workers who worked in the HIV programme in health-care facilities, and comprised doctors, nurses and midwives. Implementation first began in Jakarta in January 2019 and then in Denpasar and Semarang in July 2019. Throughout the pilot implementation period, there were no national guidelines for aPN, and service delivery varied by site and setting.

Between October and December 2018, there were 327,282 PLHIV and 46,650 new HIV infections reported in Indonesia, including Jakarta, Denpasar, and Semarang. In 2018, Jakarta reported 3763 new HIV-positive cases, Semarang reported 321 new cases, and Denpasar reported 729 new cases. During this period, the three cities reported a total of 18 serodiscordant couples with new HIV-positive diagnoses [11].

Definition of aPN referral methods generally followed WHO guidelines [14]: (i) patient referral, where PLHIV or clients themselves notify their partner of potential HIV exposure, usually including disclosure of HIV status; (ii) provider referral, where the health provider contacts partners to notify them of potential HIV exposure without disclosing the source of exposure or clients’ identity; (iii) contract referral, where a contract is made between client and provider for the client to notify the partner of HIV exposure within a certain deadline, failing which the provider would contact and notify the partner as in a provider referral; (iv) dual referral, where the client and partner arrange for a tripartite meeting at which the client would notify the partner of HIV exposure with support from the provider.

### Data collection

We conducted a formative qualitative study between September and October 2019 in three Indonesian cities where aPN was being piloted; Jakarta, Semarang, and Denpasar. Six focus group discussions (FGDs) were conducted: three with providers, two with a PLHIV peer support group, and one with the general population. Each FGD with providers and the PLHIV peer support group consisted of 8 participants, while the FGD with the general population had 14 participants. Details of FGD locations and number of participants, as well as sex distribution, are presented in Table 1. Health-care providers were recruited by the city health officer; PLHIV participants were recruited by health workers at the health facilities; and those from the general population were recruited by the local community leader. All enrolled participants were ≥ 18 years of age and consented to participating in the study.

**Table 1:** Stakeholders participating in the qualitative study from each of the demonstration sites

Location	Participants	Male	Female
Denpasar, Bali	Health-care providers	2	6
	PLHIV	2	6
	General population	11	3
Semarang, Central Java	Health-care providers	1	7
	PLHIV	7	1
Jakarta	Health-care providers	2	6

PLHIV: people living with HIV.

Providers were eligible for FGDs if they were trained in and actively providing HIV testing services and aPN in a health facility for at least three months. PLHIV were eligible for FGDs if they were members, for at least three months, of an active support group that included members of key population groups and were already familiar with the aPN programme. For example, PLHIV participants in Semarang were predominantly men who have sex with men (MSM) while in Denpasar they were a mixture of heterosexual persons and female sex workers (FSW). People from the general population were randomly selected from a *banjar,* a sub-village community in Denpasar, through open invitation. The invitation was distributed by the *banjar* leader or a *kelihan* in the local online messages group, and the same person then arranged the FGD for this group.

All FGDs were conducted by two Indonesian researchers experienced in qualitative methods, one male and one female. They used semi-structured interview guides (see Additional file 1), and audio-recorded the interviews. Consent for audio-recording was obtained beforehand. FGDs were held for no more than two hours. At the beginning of each FGD, participants were briefed on what aPN is, its purpose, and its different referral methods. All FGDs were conducted in a face-to-face, confidential setting in a meeting room at local health authorities’ office. FGDs were conducted in the Indonesian language. Both researchers had experience in working with key population groups. All recordings were transcribed in Indonesian.

Participants were asked about their experience with and exposure to the aPN programme and what aPN method they would prefer, especially the mode of initial contact, messages to be delivered when contacting partners, how to respond to questions from partners, and their rationale for each of these answers. The guides were tailored towards different stakeholder groups. FGDs among the general population included a brief explanation of aPN in case participants were previously unfamiliar with these services. All FGDs were considered complete after saturation was reached, which was determined when participants were no longer providing new information.

### Data analysis

We used thematic analysis [20] to analyse the FGD results; the data were processed in NVivo 11 (QSR International Pty Ltd). Phrases and words uttered by FGD participants were coded as they occurred in the conversation without a prior set of predetermined codes. Transcripts were first read by GBSW and coded inductively with a set of codes developed organically from the transcripts. Codes was then grouped by theme, then triangulated by study location and participant type. This inductive approach, rather than a deductive one with a predetermined set of codes of expected phrases and themes, strengthened the internal validity by reducing investigators’ prejudices in the analysis of qualitative results [20]. Internal validity was further strengthened by triangulating the themes observed by different populations and in the different cities [21]. Study findings were de-identified and aggregated to protect participant confidentiality and then disseminated to local stakeholders and the Indonesian Ministry of Health, which had no authority to edit or alter the content of the report. Individual study participants, however, did not review the results.

### Ethical considerations

Ethical approval was obtained from the Research Ethics Committee Faculty of Medicine, Udayana, University/Sanglah Hospital, Denpasar (2744/UNI14.2.2.VII.14/LP/2019). All participants provided written or witnessed informed consent to participate in FGDs and were informed about the study and its aim of understanding preferences for and perspectives of aPN service delivery. No names were used in the quotes or text in this paper and consent for publication is not applicable as no individual-level data are presented.

## Results

aPN was acceptable and well-received by all participants. However, there were some variations in preference regarding how to approach PLHIV and establish contact with their partners. Providers were mainly concerned about their ability to deliver aPN services effectively, especially with respect to encouraging clients to accept aPN services and partners to test for HIV. Providers were also concerned about subsequent physical security and the implications of current laws and policies for them, while PLHIV were mainly concerned about the confidentiality of their HIV status and the potential unintended consequences on their relationships. These concerns drove provider and participant preferences toward messages that avoided mentioning HIV exposure, and using phone calls and short messages (e.g., WhatsApp, SMS, etc.) for initial contact. General population participants had substantially fewer concerns and focused on the importance of delivering effective aPN and supported more direct and immediate approaches (such as home visits) for contacting partners.

### Providers’ perspectives

Health-care providers tend to prefer patient referral aPN services led by PLHIV or dual referral, which gives the option for PLHIV and providers to contact partners together. Providers were averse to provider referral because they perceived it to be labour intensive and questioned its effectiveness. Such perceptions were driven by past experiences where they were ignored when trying to follow up and contact partners of PLHIV. Providers also admitted to lack of confidence in responding to questions from partners due to past failure.

“But that was the initial question, where did I get the phone number. I said it was just a random number, just like that. He is still healthy, does not want to come. Since then, I don't dare to contact clients. If I slip up and say the identity, it will be really bad.”

Health-care provider (male), Denpasar

Two providers in Semarang and three in Jakarta voiced concerns about current laws and policies in Indonesia regarding aPN. The concerns revolved on the potential legal jeopardy faced by providers in case their client’s identity was inadvertently revealed during provider referral, as well as how promoting compliance from index patients and test indexes. A potential breach in confidentiality could be seen as a violation of the confidentiality of medical information.

“ … It is possible that we might get sued. Maybe not criminally, but civil lawsuit for disclosing patient’s confidential information to his/her partner. Isn’t it the same? For example, we thought we are being discreet, but if the partner only had one new partner in the last year, automatically s/he will know the identity of our patient.”

Health-care provider (male), Semarang

Providers felt that initial contact via a phone call or short message was ideal due to its simplicity and the small amount of effort required. Providers also believed that phone calls and short messages were more secure and better for preventing potential breaches of confidentiality of their PLHIV clients. Two providers in Jakarta suggested combining contact methods: starting the initial contact with a short message via text, email or social media, and then moving on to a phone call if the partner failed to respond.

“We tried using WA [WhatsApp] first. The problem is if you get a phone call sometimes the partner would come, like what I just said, [the partner] might be in the office or wherever. If the WA [short message] isn’t replied [to], then we try to call and usually it says the telephone number you are calling cannot be reached. It means the phone can't connect.”

Health-care provider (female), Jakarta

Providers generally preferred messages that did not explicitly mention HIV exposure, especially during initial contact, because they felt most partners would react negatively and it would lead to loss to follow up due to denial. Other providers noted that dual referral and aPN messages talking about HIV were important as they provided an opportunity for PLHIV to disclose their HIV status to their partner.

“I don’t say HIV, because if I do, they will run away.”

Health-care provider (female), Denpasar

“In Puskesmas (public health centre), in the beginning we don’t say HIV. We only say that your partner is having virus and in order to ensure whether you have it or not, let us test you. If the result is positive, our patients are mostly willing to open up about their status, because their partners are also positive. If the [partner’s] result is negative, then patients don’t want to disclose [their HIV status]. Then we only tell their partner ‘you don’t have the virus, but it would be great for you to come back to test every 3 months’.”

Health-care provider (female), Semarang

### PLHIV perspectives

Like providers, PLHIV were concerned about aPN and how breaches in confidentiality might affect them and their relationships with their partners. These concerns were driven by beliefs that disclosure was a personal matter and if the health worker informed their partner, it would threaten trust and the future of the relationship.

“From my partner, we already married so we trust each other. If I know [get notified] from another person, it feels like I am cheated. So it would be better if it is our partner who notifies us, slowly.”

PLHIV (female), Semarang

PLHIV participants also preferred phone calls with a message that did not explicitly mention HIV exposure as the ideal way to initially contact partners. Follow up using a face-to-face meeting (e.g., home visit) for HIV testing was considered acceptable as well if the phone calls or messages were not returned. Dual referral was also supported and highlighted as an opportunity to disclose the HIV status to their partner.

“For me, I still [prefer] via telephone first. Maybe the person is willing to make time, we can [do] direct home visits. That’s my opinion anyway.”

PLHIV (female), Denpasar

“For me, I’ll be more comfortable if told directly that I can come to the service. ‘We have something to say,’ deliver it like that. Now when we come to the service, the counsellor could help me tell my partner that I am HIV positive.”

PLHIV (female), Denpasar

### General population’s perspective

People in the general population felt that aPN was important and that patient referral would be difficult for PLHIV to implement due to stigma and concerns about their relationship. However, all participants explicitly or tacitly acknowledged the importance of PLHIV disclosing their status to their partners and encouraging HIV testing.

“Being brave to also mention the problem so that the partner is not infected. It’ll ensure partner safety. Even though it means we are infected. Daring to be brave I guess is hard, but [by that point] we’ve been infected anyway so definitely have to [tell the partner]. Not just to partners, but to parents as well.”

General population (male), Denpasar

Consequently, general population participants preferred direct and provider referral approaches for contacting partners, such as home visits or other face-to-face meetings, including dual referral. This preference was driven by perceptions that phone calls and short messages without mention of HIV exposure would not be returned and would be less effective. They highlighted that messages that directly mention HIV exposure are harder to ignore and necessary to increase awareness of the need to elicit further action, such as HIV testing.

“In my opinion, a phone call is good. But there is the possibility of a misunderstanding, like … it [would] require more communication with my husband because it may cause suspicion [and] he may question who is calling me. And if he suspects me having this disease [HIV], it would also be bad. It would harm communication with a partner. That’s why, if possible, home visits would be better than phone calls.”

General population (female), Denpasar

“For me it is better to say HIV. We know the disease and [if notified of a potential exposure] would immediately test. If we don’t really know and get invited to test, most of the time we will not be motivated to go.”

General population (male), Denpasar

## Discussion

Our study has provided new insights into the perceptions and preferences of relevant stakeholders regarding service delivery of aPN. These findings could help to inform and understand how best to tailor aPN services in settings like Indonesia. While previous studies have gathered values and preferences regarding aPN [22], this is one of the few to triangulate perceptions and preferences from three sources – PLHIV, health-care providers and the general population. A previous study on aPN in Indonesia was limited to services for the partners of incarcerated populations [23].

There was some consensus on preference for dual referral approaches, where providers and PLHIV notify partners together. However, participants from each of the groups were driven by different concerns, such as confidentiality and social stigma among PLHIV, safety among health-care providers, and effectiveness among the general population.

Confidentiality is a primary cause for concern among PLHIV. It is a common barrier to engaging in aPN services and drove preferences for patient referral methods in our study. Disclosure was considered highly personal, and there were concerns that notification without the presence of the index patient may be harmful to the relationship. Partners also expressed greater appreciation toward PLHIV who candidly informed them of their HIV status [22]. These values were linked to preferences for dual referral approaches among PLHIV. Correctly performed, many felt dual referral would maintain intimacy between partners while also providing education and mitigating the risk of negative reactions.

PLHIV and health providers were particularly concerned about potential social stigma, including potential self-stigma. This has been previously observed across aPN studies [24–26] and may be more common among populations and settings with tight-knit social structures [27], as in Indonesia, and among those that experience discrimination [23]. To address this fear, providers should consider ways in which to reassure their clients and suggest strategies to address their concerns, such as offering to send short messages or a phone call, which was often preferred for initial contact and perceived to be more confidential than other methods [28].

Providers had similar preferences for patient referral as PLHIV but were motivated by concerns for their clients’ and their own security. Most providers lacked confidence in their ability to communicate with PLHIV and their partners and discuss aPN. This was largely due to uncertainty regarding how to handle partners’ negative reactions to learning of HIV exposure, and that they may be pressured to or accidentally share clients’ identifying information. These concerns have been reported elsewhere and driven preferences for patient referral models [29, 30], especially in settings where aPN is new and offered in a setting with restrictive regulations. Such gaps highlight the importance of training, mentorship, support and supervision for health workers, especially those working in programmes that are just beginning to implement services [31].

Perspectives of participants from the general population differed from those of providers and PLHIV. They preferred provider referral methods and home visits by health-care providers, which they felt were efficient, effective and would lead to a more immediate explanation and direct notification of an HIV exposure. As a result, many participants from the general population disliked methods that they felt were less personal such as invitation letters, emails, or short messages [32]. This finding was in agreement with previous evidence. Provider-assisted referral methods, especially if immediately offered, are more effective in eliciting tests and finding new cases when compared to unassisted ones [33, 34]. Results from Kenya noted the difference immediacy brings as two third of partners of index patients who were immediately offered partner services had an HIV test, compared to less than a quarter in the delayed group [33].

All participants described the potential benefits of using digital approaches either as a replacement for or in addition to face-to-face follow up. Considering the COVID-19 context, telemedicine and follow up via mobile phones and social media are increasingly gaining importance for service delivery, including aPN [35]. Many programmes are also using HIV self-testing to further expand aPN services and have been particularly effective in reaching male partners [36]. While provider-assisted referral continues to be the most effective way of reaching partners of PLHIV [33], it is essential to provide a mix of methods that are acceptable to and suitable for clients and their partners. Personalization, according to index patients’ situation and their understanding of their partners, is important in selecting the partner notification method to be used [37].

### Research and practice implications

Dual referral approaches, which were perceived to be both a direct and confidential way to contact partners, appeared acceptable to all stakeholders, despite their differing concerns. While the approach is not unique to Indonesia, few studies have prioritized or specifically evaluated this approach. Further operational research is needed on how this method can be successfully adopted and scaled up in Indonesia.

Future programmes, which would be developed with the inputs obtained from this study, should consider these concerns and preferences when planning and implementing aPN services in Indonesia and similar settings. Greater efforts are needed train health workers so that they can gain confidence in speaking with PLHIV clients and partners about aPN options, as well as strengthening their skills to support disclosure, protect confidentiality, identify social harm and gender-based violence, and refer clients to support services.

Public health messaging is also needed to address misconceptions about HIV and HIV-related stigma (including self-stigma among PLHIV). Basic public health messaging should address misconceptions on HIV transmission in Indonesia. A considerable proportion of Indonesians still believe be transmitted by mosquito bites or sharing utensils with a PLHIV [38]. Furthermore, it should promote information about the benefits of early HIV testing, prevention and treatment, including that PLHIV on ART who are virally suppressed cannot transmit HIV to their partners. It remains critical for all aPN services to be voluntary. Mandatory HIV testing or notification is not recommended [39].

Moreover, the legal standing of aPN services offered by health-care providers should also be clarified to both providers and clients in Indonesia. To date, there is no national- or ministerial-level regulation that explicitly addresses aPN services. Oft-cited legal protection includes Minister of Health Regulation no. 21 of 2013, Article 21, which concerns early detection of HIV, and Minister of Health Regulation no. 36 of 2012 on Medical Secrecy, Article 9, which provides that confidential medical information may be disclosed in case of “threat to others’ individual or communal safety”. At the same time, there is a lack of awareness among providers of the existence of these legal umbrellas, making the perceived legal protection to be even less than it is. A codified policy that explicitly addresses aPN and its implementation is necessary.

### Limitations

While the study has many strengths and provides insights into perceptions of aPN among providers, patients and the general population in Indonesia, it focused on three cities and the findings may not be generalizable to all settings or rural areas within Indonesia. There was also gender and sexual orientation imbalance among participants in all stakeholder groups which may have affected the themes that emerged during the discussion.

Additionally, the study took place during an ongoing pilot of aPN prior to the development of clear national guidelines. This may have affected the implementation of services across sites and data collected. Also, since we relied on routine national programme data at government clinics during the study, data on actual instances of stigma, violence, or other harms as a result of aPN were not collected from among our participants.

## Conclusion

A mix of effective aPN strategies is needed to increase access to and uptake of testing among partners of PLHIV in Indonesia. Providers and PLHIV in Indonesia have similar aPN preferences, i.e. for client and dual referral models and the use of phone calls and short messages, which were perceived to minimize negative reactions and stigma, protect client confidentiality and would be suitable in the current legal situation. These preferences differed from those of the general population, which valued more direct, in-person communication of possible HIV exposure, access to HIV testing and provider referral. Scaling up and expanding these aPN strategies to serve PLHIV and their partners is critical for Indonesia and for achieving national and global goals.

## Supplementary Information

**Additional file 1.** Interview Guide. This file contains the semi-structured interview guide used in focused group discussion for data collection of this study.

## Data Availability

All data generated and/or analysed in this study are available from the corresponding author on reasonable request.

## Abbreviations

aPN: Assisted partner notification
ART: Antiretroviral therapy
FGD: Focus group discussion
FSW: Female sex worker
MSM: Men who have sex with men
PLHIV: People living with HIV
WA: WhatsApp
