# Supplementary material for: Perspectives on voluntary assisted partner notification among providers, people with HIV and the general population in Indonesia: a formative qualitative study
Source: BMC Public Health. 2021 Jan 30;21:254. doi: 10.1186/s12889-021-10332-8 (PMC7847236; doi:10.1186/s12889-021-10332-8)
Supplement: Supplementary file 1 — Additional file 1. Interview Guide. This file contains the semi-structured interview guide used in focused group discussion for data collection of this study. [file 12889_2021_10332_MOESM1_ESM.docx]

## FGD Guidelines

Target: Healthcare providers (doctors, nurses, program officers)

1. Can you tell us about how the PN program was implemented in the health facility where you work?
2. Are there rules or policies as the basis for implementing the PN program in your area / place of duty?
   1. Is it derived in SOP, service flow etc.?
   2. Is the order / SOP / service flow being socialized to all health workers / staff on duty?
   3. What is the understanding of the staff in charge of PN procedures / SOPs / flow of services?
3. Do you think you have the ability / skills in implementing PN? Why is that? What about other officers?
   1. How was the PN implementation officer appointed? Who is pointing?
   2. Is there training before health workers carry out PN? Who gives?
   3. What is the training like? What Material? Is it considered sufficient to be able to implement PN?
   4. Are there materials / knowledge that need to be given to officers? Why is that?
4. At the service where you are assigned, what PN model is implemented? (patient / officer / double / contract / community)
   1. Is there a basis for the selection?
   2. What is the level of success?
   3. Are there different models of PN implementation in various key populations? Like what? Why is that?
5. When does the officer usually introduce the PN program to the client? Why is that?
   1. When do you think is the right time to introduce the PN program to clients? Why is that?
   2. How has the client's response been offered by PN? (pro vs cons) Why did it happen?
6. What specific method do you usually use to notify client pairs? Why is that? (meet in person / phone / email / SMS etc.)
   1. Does the method succeed / fail? Why is that? What is the acceptance of the index test for this method?
   2. In implementing this method, do you use guidelines from the Ministry of Health? Why is that?
   3. Are the sample words provided in the MOH guidelines in accordance with practice on the ground? (non-discriminatory, non-enforcing, etc.). Why is that? How should it be?
   4. In your opinion, what specific method would best be used to notify a client partner? Why is that?
7. Are there specific strategies for implementing PN to different key populations? Like what? Why is that?
8. So far have there been any adverse effects caused by the implementation of the PN? Like what? Why is that? (prob: Confidentiality, domestic problems, partner violence, etc.)
   1. If it happens, what treatment will the officer take to deal with it? Why is that?
   2. Was it successful / failed? Why is that?
   3. Who is involved in overcoming the adverse effects of the PN? Who else should be involved? Why is that?
9. During PN implementation, what do you think is the motivation that makes clients willing to join PN? Why is that? Are they the same / different in each key population?
10. So far, other than those mentioned above, what are the obstacles that prevent clients from engaging in PN programs?
    1. Are there distance / or transportation barriers?
    2. Are there financing constraints?
    3. Are there social / community barriers?
    4. Are there other obstacles?
    5. How have you been overcome these obstacles?
11. What is usually done for PLWHA who do not want to receive PN?
    1. What should be done to PLWHA who do not want to receive the PN program, due to various reasons and obstacles?
    2. What is the role of health workers, health offices, NGOs / CBOs and peer support groups to initiate PN in ODHA who refuse?
12. In your view, what changes do you feel need to be made, to improve PN implementation? Why is that?
13. During this time in the overall PN implementation, which parties / sections (in health facilities) were involved?
    1. Is there a referral flow? Like what? Is it effective? Why is that? What should be done to improve the referral flow?
    2. Does every part / poly understand PN alignment flow?
    3. What about reconciliation outside health facilities? Who is involved? Is it effective?
14. So far, how has the PN reporting process been carried out?
    1. Who made the reporting? Are there any difficulties? Why is that?
    2. To whom was the report given?
    3. Is the reporting process in accordance with what is determined? If not, why?
    4. Is there feedback from the Health Office regarding the reports collected? Why is that?
    5. Do you evaluate the implementation by using the PN implementation check list at the Health Office developed by the Ministry of Health? How far has it been used? Is it required in filling it? How to follow up?
15. During this time, has there been supervision and monitoring in the implementation of PN?
    1. Who did it? How often? What kind of monitoring activities?
    2. What things are usually monitored?
    3. How have the results of monitoring been utilized by the Health Office?
16. So far, has there been an evaluation of the PN program's ignition?
    1. Who organized?
    2. How often? (month / quarter / year). How often should the evaluation be done? Why is that?
    3. Are staff involved in the evaluation activities? If yes, what kind of involvement? If not, why not be involved, how should it be?
    4. In your opinion, have the PN evaluation activities been effective? (or help improve program performance)? Why is that? What should be done?
    5. How have the results of the evaluation been utilized by the Health Service?
17. In the implementation of PN, did the presence of peer support groups help? Why is that? What should happen?
    1. In your view, what is the role of peer support groups in implementing PN?
    2. What are the strategies for increasing the effectiveness of peer support groups?
18. In the implementation of the PN, what support was felt to be sufficient? Is there anything that is not enough?
    1. What about leadership and managerial?
    2. What about facilities and infrastructure?
    3. How about teamwork?
    4. Are there other supports needed but not yet provided?

## FGD Guidelines

Target: PLHIVs in peer support group

1. Can you tell how this peer support group was involved in the implementation of the PN program?
2. Are there rules or policies as the basis for involving peer groups in implementing PN programs? Is it socialized to all members of peer groups? Why is that?
3. In your view, what is the role of peer support groups in implementing PN?
4. What are the strategies for increasing the effectiveness of peer support groups?
5. What do you think about the PN program? Does it help in HIV detection? Why is that?
6. During this time, what do you think about the implementation of the PN program? Is it effective? Why is that? What should be improved?
7. When does the officer usually introduce the PN program to the client? Why is that?
   1. When do you think is the right time to introduce the PN program to clients? Why is that?
   2. How has the client's response been offered by PN? (pro vs cons) Why did it happen?
8. What specific method do you usually use to notify client pairs? Why is that? (meet in person / phone / email / SMS etc.)
   1. Does the method succeed / fail? Why is that? What is the acceptance of the index test for this method?
   2. In implementing this method, do you use guidelines from the Ministry of Health? Why is that?
   3. Are the sample words provided in the MOH guidelines in accordance with practice on the ground? (non-discriminatory, non-enforcing, etc.). Why is that? How should it be?
   4. In your opinion, what specific method would best be used to notify a client partner? Why is that?
9. Are there specific strategies for implementing PN to different key populations? Like what? Why is that?
   1. What PN model should be applied to these different key populations?
10. So far have there been any adverse effects caused by the implementation of the PN? Like what? Why is that? (prob: Confidentiality, client's household problems, etc.)
    1. If it happens, what treatment will the officer take to deal with it? Why is that? What kind of treatment is KDS doing?
    2. Was it successful / failed? Why is that?
    3. Who is involved in overcoming the adverse effects of the PN? Who else should be involved? Why is that?
11. So far, has there been monitoring / evaluation of PN program ignition?
    1. Who organized?
    2. How often? (month / quarter / year). How often should monev be carried out? Why is that?
    3. Are peer support groups involved in evaluation activities? If yes, what kind of involvement? If not, why not be involved? how should
    4. Is it involved in assessing implementation using a checklist of PN implementations at the Fasyankes developed by the Ministry of Health? How far has it been used? Is it required in filling it? How to follow up?
    5. In your opinion, have the PN evaluation activities been effective? (or help improve program performance)? Why is that? What should be done?
12. In the implementation of the PN, what support was felt to be sufficient? Is there anything that is not enough?
13. During PN implementation, what do you think is the motivation that makes clients willing to join PN? Why is that? Are they the same / different in each key population?
14. So far, other than those mentioned above, what are the obstacles that prevent clients from engaging in PN programs?
    1. Are there distance / or transportation barriers?
    2. Are there financing constraints?
    3. Are there social / community barriers?
    4. Are there other obstacles?
    5. How have you been overcome these obstacles?
15. What is usually done for PLWHA who do not want to receive PN?
    1. What should be done to PLWHA who do not want to receive the PN program, due to various reasons and obstacles?
    2. What is the role of health workers, health offices, NGOs / CBOs and peer support groups to initiate PN in ODHA who refuse?
16. In your view, what changes do you feel need to be made, to improve PN implementation? Why is that?
